# *EGFR*, *KRAS*, *BRAF*, *ALK*, and *cMET* genetic alterations in 1440 Sardinian patients with lung adenocarcinoma

**DOI:** 10.1186/s12890-019-0964-x

**Published:** 2019-11-11

**Authors:** Maria Colombino, Panagiotis Paliogiannis, Antonio Cossu, Davide Adriano Santeufemia, Sardinian Lung Cancer (SLC) Study Group, Antonio Pazzola, Giovanni Maria Fadda, Pietro Pirina, Alessandro Fois, Carlo Putzu, Giorgio Ginesu, Alberto Porcu, Giorgio Astara, Mario Scartozzi, Anna Maria Carta, Efisio Defraia, Daniela Guerzoni, Giuseppe Porcu, Gianfranco Bardino, Claudio Sini, Francesca Capelli, Maria Giuseppina Sarobba, Maria Cristina Sini, Milena Casula, Grazia Palomba, Antonella Manca, Marina Pisano, Valentina Doneddu, Giuseppe Palmieri

**Affiliations:** 1 Unit of Cancer Genetics, Institute Biomolecular Chemistry, CNR, Traversa La Crucca 3, 07100 Sassari, Italy; 2 Department of Medical, Surgical, and Experimental Sciences, University of Sassari, Viale San Pietro 43, 07100 Sassari, Italy; 3 Medical Oncology Unit, Civil Hospital, Via Don Minzoni, 07041 Alghero, Italy

**Keywords:** Lung cancer, Adenocarcinoma, Targeted therapies, *EGFR*, *KRAS*, *BRAF*, *ALK*, *cMET*

## Abstract

**Background:**

Lung cancer is one of the most incident neoplastic diseases, and a leading cause of death for cancer worldwide. Knowledge of the incidence of druggable genetic alterations, their correlation with clinical and pathological features of the disease, and their interplay in cases of co-occurrence is crucial for selecting the best therapeutic strategies of patients with non-small cell lung cancer. In this real-life study, we describe the molecular epidemiology of genetic alterations in five driver genes and their correlations with the demographic and clinical characteristics of Sardinian patients with lung adenocarcinoma.

**Methods:**

Data from 1440 consecutive Sardinian patients with a histologically proven diagnosis of lung adenocarcinoma from January 2011 through July 2016 were prospectively investigated. *EGFR* mutation analysis was performed for all of them, while *KRAS* and *BRAF* mutations were searched in 1047 cases; *ALK* alterations were determined with fluorescence in situ hybridization in 899 cases, and cMET amplifications in 788 cases.

**Results:**

*KRAS* mutations were the most common genetic alterations involving 22.1% of the cases and being mutually exclusive with the *EGFR* mutations, which were found in 12.6% of them. *BRAF* mutations, *ALK* rearrangements, and *cMET* amplifications were detected in 3.2, 5.3, and 2.1% of the cases, respectively. Concomitant mutations were detected only in a few cases.

**Conclusions:**

Almost all the genetic alterations studied showed a similar incidence in comparison with other Caucasian populations. Concomitant mutations were rare, and they probably have a scarce impact on the clinical management of Sardinians with lung adenocarcinoma. The low incidence of concomitant *cMET* amplifications at diagnosis suggests that these alterations are acquired in subsequent phases of the disease, often during treatment with TKIs.

## Background

Lung cancer is one of the most incident neoplastic diseases and a leading cause of death for cancer worldwide [1, 2]. Its incidence has been increasing in developing countries and in women in the last decade, while it began to decline in males in most developed countries [2]. Mortality rates remain high, despite recent advances in the prevention, screening, surgical and medical management of patients with lung cancer. Surgery is an effective treatment in the early stages of the non-small cell lung cancer (NSCLC) subtypes; unfortunately, approximately 80% of the sufferers are at an advanced stage at the time of diagnosis, and approximately 20% of them are affected by small cell lung cancer (SCLC), which has no substantial benefits from surgery [3]. Chemotherapy has been the main treatment available for advanced stage patients for years. Last-generation chemotherapy drugs combined with a platinum regimen showed a 5-year survival improvement of 11%, but with a median survival time of only 8–10 months [4, 5]. In addition, chemotherapy drugs cannot differentiate tumour cells and normal cells, leading to dramatically strong adverse reactions that compromise the effectiveness and completeness of therapies.

Efforts to improve the results of the oncological treatments for NSCLC, together with the technological advances in DNA sequencing, led to the development of new therapeutic strategies based on the knowledge and classification of specific molecular features of the disease. Subsets of patients with adenocarcinoma and activating mutations within the kinase domain of the epidermal growth factor receptor (*EGFR*) gene have been successfully treated with selective tyrosine kinase inhibitors (TKIs), such as erlotinib, gefitinib, afatinib, and osimertinib, which are also characterized by reduced adverse events in comparison with traditional chemotherapy [6, 7]. In addition, anaplastic lymphoma kinase (ALK) and ROS proto-oncogene 1 (ROS1) fusions have been demonstrated to be effectively druggable with targeted inhibitors such as crizotinib, alectinib, and ceritinib and are currently recommended for the treatment of advanced stage adenocarcinoma harbouring that kind of genetic alteration [8–10]. Furthermore, active research is ongoing for the evaluation of the clinical impact of additional draggable genetic alterations, such as Kirsten rat sarcoma viral oncogene homologue (*KRAS)* and v-raf murine sarcoma viral oncogene homologue B (*BRAF)* mutations or proto-oncogene *cMET* amplifications involved in the pathogenesis of lung cancer, and have proven effective in treating other malignancies [11, 12]. In addition, the coexistence of driver mutations in the same tumours has been demonstrated to consistently impact the therapeutic outcomes and survival rates of patients undergoing chemotherapy or targeted therapies for NSCLC, as they can alter the responses to target therapies [13]. For these reasons, recent guidelines suggest that 9 genes related to targeted therapies should be detected, including *EGFR*, *KRAS*, *HER2*, *ALK*, *ROS1*, *cMET*, *BRAF*, *RET*, and *NTRK* [14].

Knowledge of the incidence of such genetic alterations, their correlation with clinical and pathological features of the disease, and their interplay in cases of co-occurrence is crucial for selecting the best therapeutic strategies of patients with NSCLC. In the present study, we describe the molecular epidemiology of *EGFR, KRAS, BRAF, ALK* and *MET* genetic alterations and their correlations with the demographic and clinical characteristics of 1440 Sardinian patients with lung adenocarcinoma.

## Methods

### Patients and samples

A total of 1440 consecutive Sardinian patients with a histologically proven diagnosis of adenocarcinoma of the lung from January 2011 through July 2016 were prospectively enrolled and investigated. For all the patients enrolled, medical records and pathology reports were used to retrieve the demographic and clinical data at the time of diagnosis; sex, age, smoking habits, type of sample (primary tumour or metastasis) and the origin of the sample (biopsy or surgery) were assessed. To avoid any bias, the patients were consecutively enrolled regardless of age at diagnosis and disease characteristics of the primary tumour. Sardinian origin was ascertained through verification of the place of birth for all patients. All patients were informed about the aims of this study and, before the tissue sample was collected, provided written informed consent. The study was performed in accordance with the principles of the Declaration of Helsinki and was approved by the Committee for the Ethics of the Research and Bioethics of the National Research Council (CNR).

### Molecular analyses

**Fig. 1:**
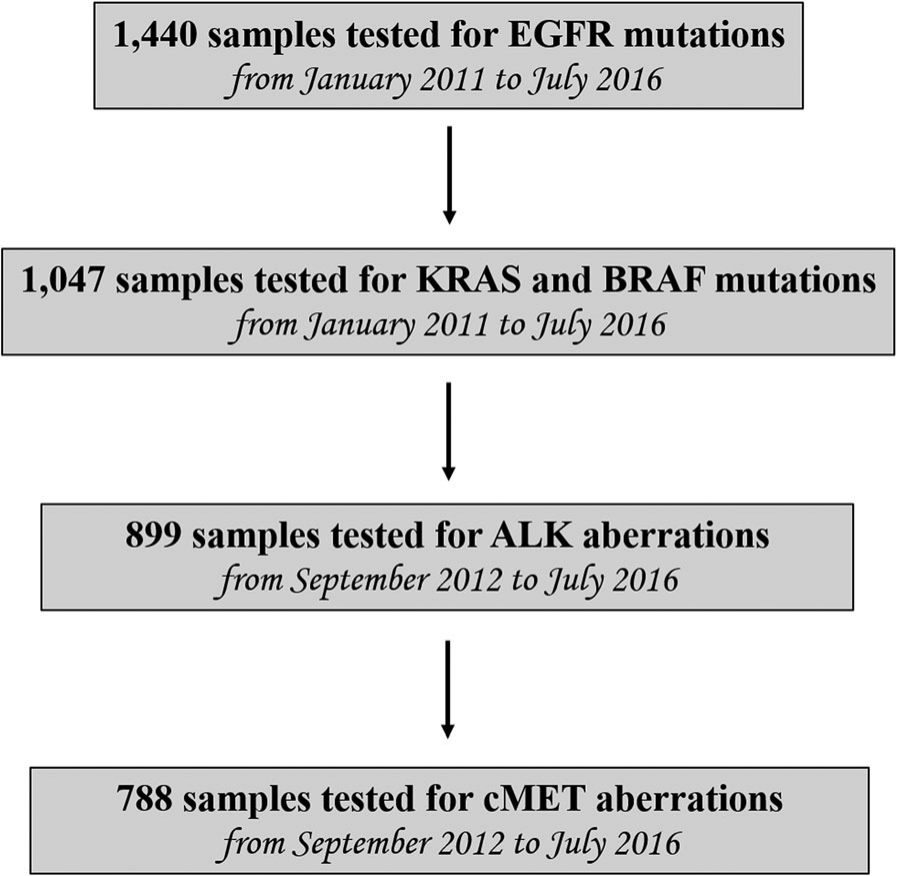
Flow chart summarizing the genetic tests performed in the study

Formalin-fixed, paraffin-embedded lung adenocarcinoma tissue samples from each patient were obtained from the Institutes of Pathology participating in the study. Tissue sections were estimated by light microscopy to contain at least 80% of neoplastic cells. In cases with lower neoplastic cell content, tissue sections (placed on slides) underwent tumour macro-dissection (using a single edge razor blade and a marked haematoxylin/eosin slide as a guide) to remove unwanted tissue parts and enrich the specimen with malignant cells. All tumour tissues were processed at the Institute of Biomolecular Chemistry (CNR, Sassari, Italy), which performed molecular analyses for all the Sardinian hospitals in the period of the study. *EGFR* mutation analysis was performed in all cases, as it was the first to be introduced in clinical practice. *KRAS* and *BRAF* mutation analysis was started subsequently and was carried out globally in 1047 cases with available biopsy tissue. The study of the genetic alterations of *ALK* started in September 2012 with the introduction of the test in clinical practice and involved 899 patients. Finally, *cMET* amplification testing was carried out in 778 cases with available tissue samples (Fig. 1).

Genomic DNA was isolated from tissue sections using a standard protocol, and DNA quality was assessed for each specimen, as previously reported [6]. Briefly, paraffin was removed from formalin-fixed paraffin-embedded (FFPE) samples by treatment with Bio-Clear (Bio-Optica, Milan, Italy), and DNA was purified using the QIAamp DNA FFPE Tissue Kit (Qiagen Inc., Valencia, CA, USA) following the manufacturer’s instructions. Yields of purified DNA were assessed by the Qubit *dsDNA High-Sensitivity Assay Kit* on the Qubit 2.0 Fluorometer (Life Thermofisher, Waltham, MA USA).

Mutation analysis was conducted in the coding sequence of the following genes: *EGFR* (exons 18, 19, and 21, where all mutations predicting the response of treatment with EGFR tyrosine kinase inhibitors are located), *KRAS* (the entire coding portion: exons 2, 3, and 4), and *BRAF* (exon 15, where nearly all of the oncogenic mutations are located). Quantitative measurements of mutations were based on pyrosequencing methodology, which is a real-time sequencing-by-synthesis approach that allows for the quantification of mutated alleles with a detection limit of 5–7% [15]. Pyrosequencing represents a good compromise between specificity and sensitivity among commonly used mutational analysis methods (Sanger-based sequencing: specificity 100%, sensitivity 15–20%; pyrosequencing: specificity 90%, sensitivity 5–7%; real-time PCR assay: specificity - for each single variant only − 100%, sensitivity 2–3%) [16]. Pyrosequencing assays were performed on a PyroMark Q24 system (Qiagen Inc., USA) following the manufacturer’s instructions.

Fluorescence in situ hybridization (FISH) analysis was carried out in interphase tumour cells using the following: for *cMET*, the specific CTB.13 N12 BAC probe (at the 7q31.2 locus) and the control centromere, labelled with Spectrum-Orange and Spectrum-Green (Vysis, Downer’s Grove, IL, USA), respectively; for *ALK*, the ALK Break Apart FISH Probe Kit (Vysis, USA). Protocols for FISH analysis were as previously described by our group [12].

**Fig. 2:**
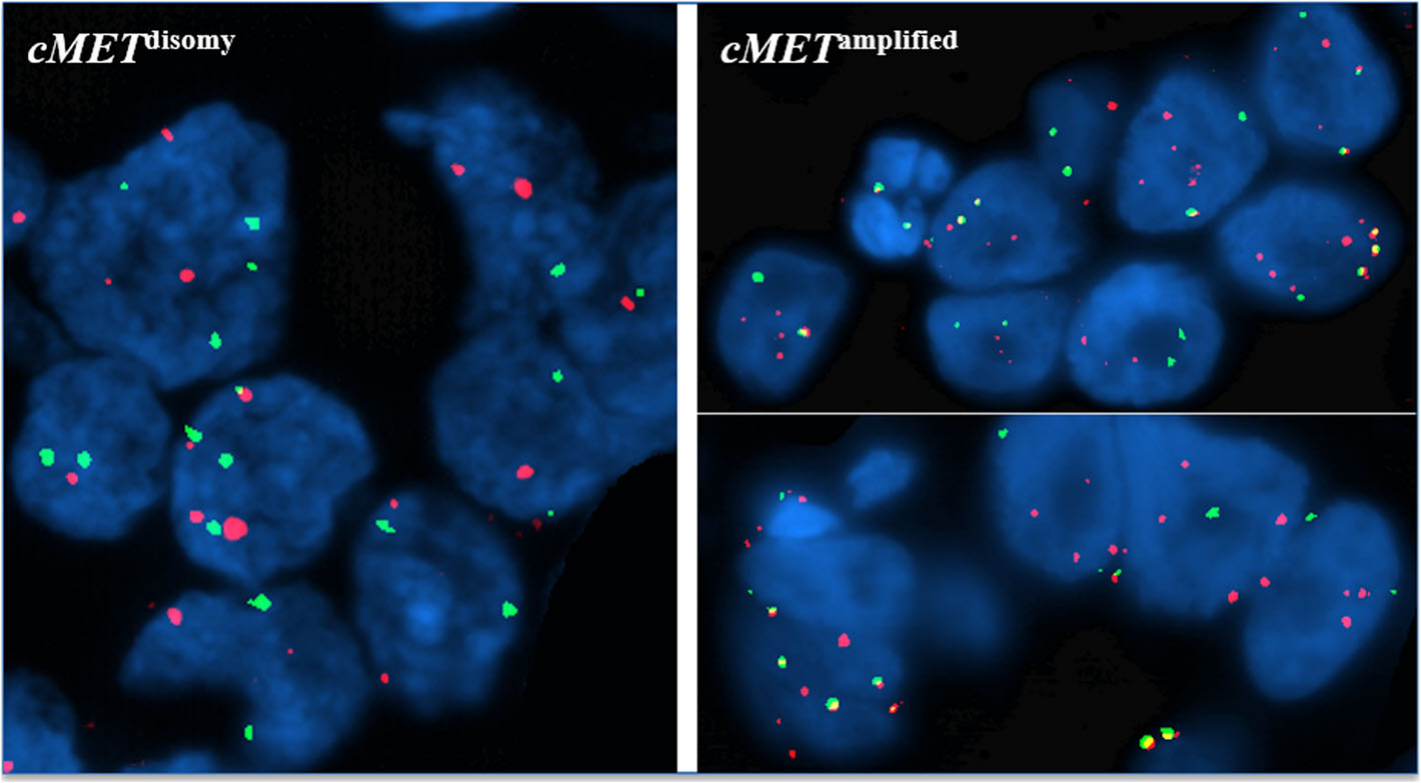
Figure illustrating cases of *cMET* disomy (left) and amplification (right)

For *ALK*, the presence of rearrangement was defined when ≥ 15% of cells were positive for FISH signals at the breakpoint of the gene at chromosome 2p23, according to the indications provided for the ALK Break Apart FISH Probe Kit (Vysis, USA). Amplification of the *cMET* gene was defined by the presence of at least one of the following criteria: *a*) candidate gene to control centromere ratio ≥ 2, according to the main criterion provided for assessing *EGFR* gene copy number in NSCLC; and/or *b*) presence of at least a tetrasomic signal (≥ 2.0 gene copies per control centromere) in more than 15% of cells. Specimens presenting none of the criteria for *cMET* gene signals were classified as disomic (Fig. 2).

### Statistical analyses

Descriptive analysis for qualitative and quantitative variables was conducted using proportions and the mean ± standard deviation (SD), respectively. Variable distribution was assessed by the Shapiro-Wilcoxon test. Statistical differences between groups were compared using unpaired Student’s t-test, Mann-Whitney rank sum test, chi-square test or Fisher’s exact test as appropriate. *P* ≤ 0.05 was considered statistically significant. Data were analysed using STATA 13® statistical software (StataCorp LP, College Station, TX, USA).

## Results

Nine hundred sixty-three (67%) of the 1440 patients enrolled were males, and the mean age was 67 (range 30–88). Most of the cases (1064, 74%) involved individuals with more than 61 years of age. Only 13% (186 cases) were never smokers, 475 (33%) were active smokers, and 538 (37%) were former smokers; data regarding smoking habits were not available in 241 (17%) cases. The samples were obtained from the primary tumour in 1243 (86%) of the cases and from metastatic lesions in the remaining cases. Finally, in only 242 (17%) cases, the specimen was obtained by surgery, reflecting the advanced stage of the disease at diagnosis in most cases in which exclusively a biopsy was performed.

The main demographic and clinical data of the patients included in the study in relation to the genetic alterations of the genes evaluated are depicted in Tables 1, 2, 3 and 4. Among the 1440 cases evaluated, 181 (12.6%) *EGFR* mutations were detected, and they were significantly more frequent in females and never smokers (Table 1). The most common mutations were L858R in exon 21 and *del*ELREA in exon 19, accounting for 38 and 29% of all *EGFR* alterations, respectively (Additional file 1: Table S1); in one case, both of these mutations occurred simultaneously.

**Table 1:** Distribution of EGFR mutations according to patients’ characteristics

Characteristics	Number of patients (%)	EGFR mutated cases		
		No.	%	*p*
Total cases analyzed	1440	181	12.6	
Sex				
Males	963 (67)	66	6.9	< 0.001
Females	477 (33)	115	24.1	
Age at diagnosis				
Median (range)	67 (30–88)			
≤ 50 years	112 (8)	20	17.9	0.107*
51–60 years	264 (18)	31	11.7	
61–70 years	544 (38)	56	10.3	
> 70 years	520 (36)	74	14.2	
Smoking habitus				
Smoker	475 (33)	17	3.6	< 0.001**
Former smoker	538 (37)	45	8.4	
Never smoker	186 (13)	109	58.6	
Unknown	241 (17)	10	4.1	
Type of sample				
Primary tumor	1243 (86)	156	12.6	0.952
Metastasis	197 (14)	25	12.7	
Sample’s origin				
Biopsy	1198 (83)	149	12.4	0.818
Surgery	242 (17)	32	13.2	

* Patients ≤ 50 years vs patients ≥ 50 years; ** Never smokers vs smokers and ex-smokers; significance at 0.05

**Table 2:** Distribution of KRAS and BRAF mutations according to patients’ characteristics

Characteristics	Number of patients (%)	KRAS mutated cases			BRAF mutated cases		
		No.	%	*p*	No.	%	*p*
Total cases analyzed	1047	231	22.1		34	3.2	
Sex							
Males	693 (66)	169	24.4	0.018	25	3.6	0.462
Females	354 (34)	61	17.2		9	2.5	
Age at diagnosis							
≤ 50 years	94 (9)	24	25.5	0.472*	3	3.2	1.000*
51–60 years	189 (18)	46	24.3		7	3.7	
61–70 years	394 (38)	78	19.9		10	2.5	
> 70 years	370 (35)	83	22.4		14	3.6	
Smoking habitus							
Smoker	336 (32)	74	22.0	0.001**	14	4.2	0.411**
Former smoker	367 (35)	89	24.3		10	2.7	
Never smoker	139 (13)	14	10.1		2	1.4	
Unknown	205 (20)	54	26.3		8	3.9	
Type of sample							
Primary tumor	889 (85)	197	22.2	0.940	29	3.3	1.000
Metastasis	158 (15)	34	21.5		5	3.2	
Sample’s origin							
Biopsy	848 (81)	193	22.8	0.304	28	3.3	0.987
Surgery	199 (19)	38	19.1		6	3.0	

* Patients ≤ 50 years vs patients ≥ 50 years; ** Never smokers vs smokers and ex-smokers; significance at 0.05

**Table 3:** Distribution of ALK rearrangements according to patients’ characteristics

Characteristics	Number of patients (%)	ALK mutated cases		
		No.	%	*p*
Total cases analyzed	899	48	5.3	
Sex				
Males	641 (67)	27	4.2	< 0.001
Females	258 (33)	21	8.1	
Age at diagnosis				
≤ 50 years	62 (8)	8	12.9	0.014*
51–60 years	168 (18)	13	7.7	
61–70 years	344 (38)	15	4.4	
> 70 years	325 (36)	12	3.7	
Smoking habitus				
Smoker	288 (33)	11	3.8	0.459**
Former smoker	332 (37)	21	6.3	
Never smoker	107 (13)	8	7.5	
Unknown	172 (17)	8	4.7	
Type of sample				
Primary tumor	783 (87)	41	5.2	0.892
Metastasis	116 (13)	7	6.0	
Sample’s origin				
Biopsy	730 (83)	39	5.3	0.850
Surgery	169 (17)	9	5.6	

* Patients ≤ 50 years vs patients ≥ 50 years; ** Never smokers vs smokers and ex-smokers; significance at 0.05

**Table 4:** Distribution of cMET rearrangements according to patients’ characteristics

Characteristics	Number of patients (%)	cMET mutated cases		
		No.	%	*p*
Total analyzed	778	16	2.1	
Sex				
Males	641 (69)	9	1.4	0.231
Females	242 (31)	7	2.9	
Age at diagnosis				
≤ 50 years	55 (7)	1	1.8	1.000*
51–60 years	139 (18)	5	2.6	
61–70 years	301 (39)	5	1.7	
> 70 years	283 (36)	5	1.8	
Smoking habitus				
Smoker	264 (33)	5	1.9	1.000**
Former smoker	295 (37)	6	2.0	
Never smoker	94 (13)	2	2.1	
Unknown	125 (17)	3	2.4	
Type of sample				
Primary tumor	661 (85)	13	2.0	0.720
Metastasis	117 (15)	3	2.6	
Sample’s origin				
Biopsy	654 (84)	13	2.0	0.730
Surgery	124 (16)	3	2.4	

* Patients ≤ 50 years vs patients ≥ 50 years; ** Never smokers vs smokers and ex-smokers; significance at 0.05

**Fig. 3:**
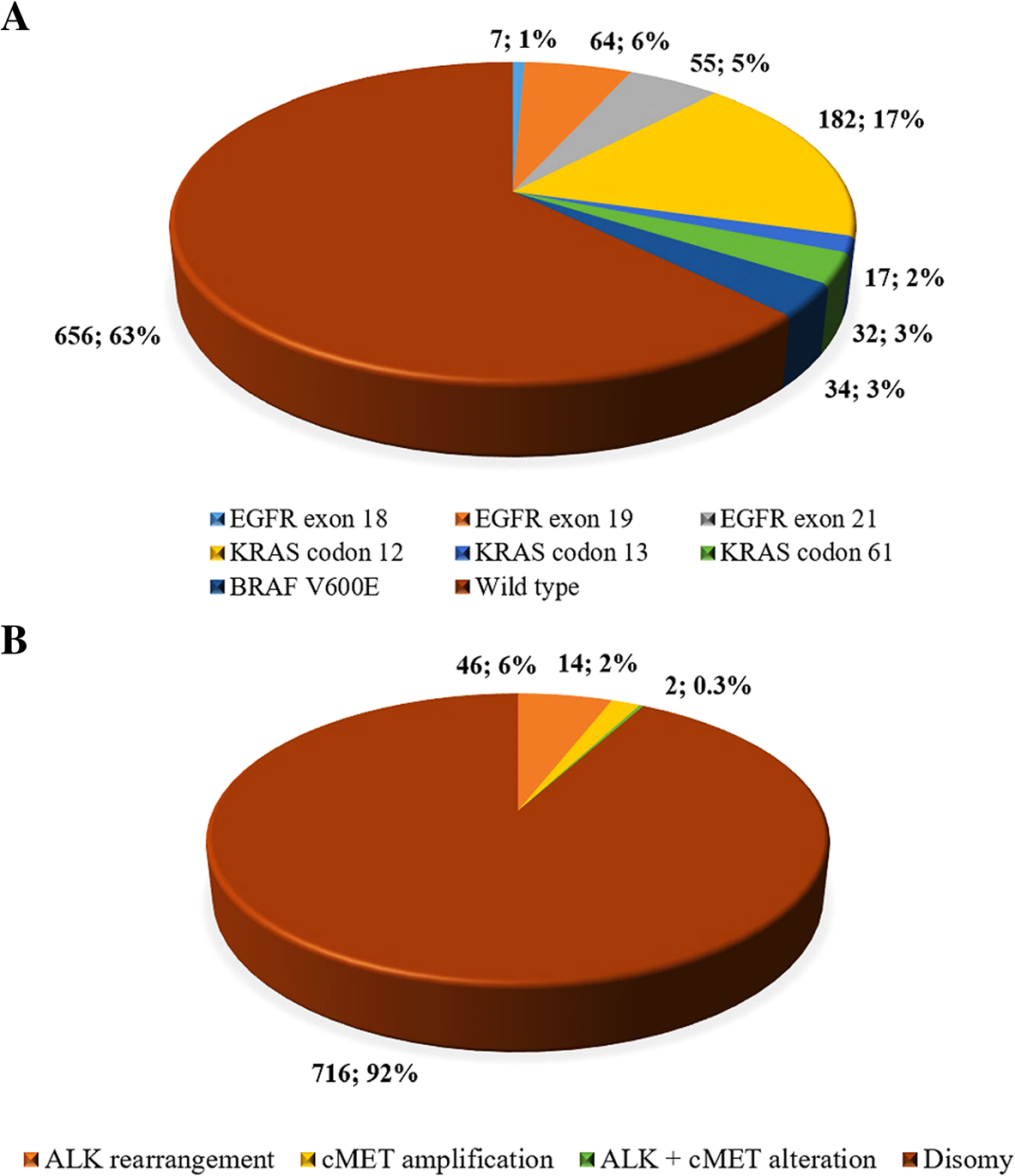
**a** distribution of the main genetic alterations among the 1047 patients tested for *EGFR, KRAS* and *BRAF* mutations. **b** distribution of genetic alterations among the 788 samples tested for *ALK* and *cMET* alterations

*EGFR, KRAS* and *BRAF* mutations were simultaneously found in a subset of 1047 patients, with approximately 37.3% of them presenting a genetic alteration in one or more of the genes examined. The most common mutations involved codon 12 of the *KRAS* gene (184, 17.4%), followed by mutations in exon 19 and 21 of *EGFR* (Fig. 3), while *BRAF* mutations were detected in 34 (3.2%) cases. *KRAS* mutations were detected in 22.1% of the examined cases and were significantly more frequent in smokers or former smokers compared to never smokers; in addition, they were significantly more frequent in males than in females (Table 2). The most common *KRAS* alterations were missense mutations in exon 2, namely, G12C (39.8%), G12 V (16.5%), and G12D (13.9%); missense mutations in exon 3 accounted globally for 13.9% (Additional file 1: Table S1). V600E in exon 15 was the only *BRAF* mutation detected in our cohort and did not show any sex or smoking habit predilection.

*ALK* rearrangements were detected in 48 (5.3%) out of the 899 cases examined; they were significantly more common in females and individuals younger than 50 years of age (Table 3). Furthermore, examining the 778 patients in which both *ALK* rearrangement and *cMET* amplification analysis were carried out, genetic alterations were found in 8% of the cases, the most common being *ALK* rearrangements (43 cases, 5.9%), while *cMET* amplifications occurred in 16 (2.1%) cases (Fig. 3). The only concomitant genetic alterations found in these patients involved two cases (0.3%) with an *ALK* rearrangement and an amplification of *cMET*, and two cases harbouring an *EGFR* mutation and an amplification of *cMET*. *cMET* amplification showed no predilection for any of the clinical parameters evaluated (Table 4). In summary, in our series, *EGFR* mutations were significantly more incident in females and never smokers, *KRAS* mutations in males and in smokers, and *ALK* rearrangements in females and individuals with less than 50 years of age.

**Fig. 4:**
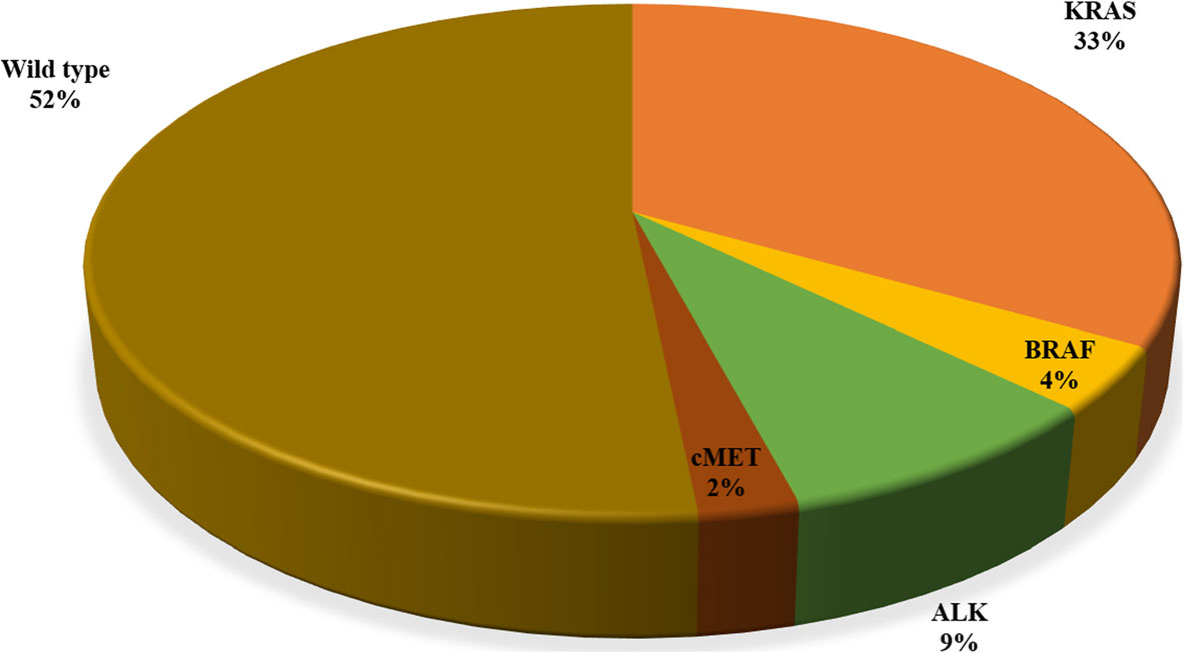
Rates of genetic alterations among the 528 patients with *EGFR* wild type tumors analyzed for alterations in all the remaining genes

Considering the 528 *EGFR* wild-type cases in which further mutational analyses were carried out, 272 (51.6%) did not present any other genetic alteration, while one-third harboured a *KRAS* mutation; percentages of alterations in the remaining *EGFR* wild-type cases are summarized in Fig. 4.

## Discussion

The study of the genetic alterations in patients with NSCLC has profoundly changed the therapeutic landscape of the disease. Considering the 1047 patients in whom *EGFR*, *KRAS*, and *BRAF* mutation analysis was simultaneously carried out in our study, approximately 37% were found to have a genetic alteration in one of the examined genes. This percentage is slightly lower than those of previous studies, which reported approximately half of the patients with lung adenocarcinoma harbouring an *EGFR* or *KRAS* mutation [17]. This may depend on the genetic characteristics of the target population in our study, characterized by high levels of genetic homogeneity due to geographical reasons. In any case, the concept that mutations in driver genes occur in a consistent percentage of lung adenocarcinomas remains, but its impact seems to be different in different populations; indeed, the incidence of *EGFR* mutations is significantly higher in Asian populations (even higher than 50%) compared to western countries [18].

In our series, *EGFR* mutations were searched in 1440 patients and were found in 12.6% of them, which is consistent with the partial results published in a previous report involving patients from the same population [6]. This figure is slightly lower than that described in recent prospective studies performed in other Caucasian populations [19, 20]. In addition, *EGFR* mutations significantly more frequent in females (24.1%) and never smokers (58.6%), a finding that has been extensively reported in previous studies and from different geographical areas [6, 19, 21]. The incidence of *EGFR* mutations has been reported as low as 28% in American never smokers and as high as 68% in Asian never smokers [22]; the rate found in our series is closer to those reported in Asian populations. As mere speculation, it is interesting that Sardinians, who have long been recognized as forming a distinct outlier within contemporary European genetic diversity, experienced an immigration of individuals belonging to the initial wave of migration from the Asian areas (mainly the Middle East) into southeastern Europe during the early Neolithic transition, leading to the observed genetic affinity of the ancients descending from these migrants to present-day Sardinians [23, 24].

A recent meta-analysis evaluated the *EGFR, ALK-EML4* and *KRAS* mutational patterns in smokers and non-smokers of various ethnicities [20]. The authors confirmed that there was a significantly increased risk of presenting *EGFR* mutations and *ALK-EML4* fusions in never smokers compared to ever smokers with adenocarcinoma. In addition, as the smoking history increased, there was a decreased risk for exhibiting the *EGFR* mutation, particularly for cases > 30 pack-years. Compared to ever smokers, never smokers had a decreased risk of *KRAS* mutations in all the populations examined [22]. Both the meta-analysis results on *EGFR* and *KRAS* mutations were confirmed in our study.

Regarding the types of the specific *EGFR* mutations, L858R in exon 21 and deletion in exon 19 were the most frequent, accounting for 38 and 29% of all the observed *EGFR* alterations, respectively. Again, these mutations are also the most frequent in studies in Asian populations, but with lower percentages [25]. Concomitant L858R mutation and deletion in exon 19 were found only in one case in our series; such a concomitance seems to be more frequent in studies in Asian populations [25]. Examining the subset of patients without *EGFR* mutations, we found that half of them had no additional genetic alteration. As expected, most of the remaining *EGFR*^wild-type^ patients harboured *KRAS* mutations (approximately one-third of the total *EGFR*^wild-type^ cases), followed by *ALK* rearrangements and to a lesser extent by *BRAF* mutations and *cMET* amplification.

*KRAS* mutations were detected in 22.1% of the cases examined, while the only *BRAF* mutation described was V600E in exon 15 detected in 3.2% of the cases examined. In a study performed at the Memorial Sloan-Kettering Cancer Center, testing of 2529 cases for *KRAS* mutations (codons 12 and 13) detected 670 (26%) mutations, including G12C (39%), G12 V (21%), G12D (17%), G12A (11%), and other G12 and G13 mutations (12%) [17]. Additionally, in our series, the most common *KRAS* alterations were missense mutations in exon 2, namely, G12C (39.8%), G12 V (16.5%), and G12D (13.9%); missense mutations in exon 3 accounted globally for 13.9% of the total. *KRAS* mutations in our series were significantly associated with male sex and smoking history of the patients, as previously mentioned [26].

Additionally, *BRAF* mutations were more frequent in males in our cohort. Involved in the RAS-MEK-ERK signalling pathway, BRAF is a serine/threonine kinase that lies downstream of RAS and has gained the most attention in malignant melanomas, where a V600E mutation is a common driver that is the therapeutic target of the selective *BRAF* inhibitors (vemurafenib, dabrafenib, encorafenib) and MEK inhibitors (cobimetinib, trametinib, binimetinib) [27]. Up to 8% of lung adenocarcinomas harboured *BRAF* mutations in recent studies (including Italian cohorts), most of them being the V600E mutation, which was the only *BRAF* alteration detected in our cohort in 3.2% of the cases examined [28–32]. Nevertheless, in a recent study performed using a next-generation sequencing approach on 36 lung adenocarcinomas, *BRAF*^V600E^ mutations occurred in 28% of the cases, mostly in smokers (90%), and in concomitance with *AKT* or *PIK3CA* mutations, non-V600E mutations occurred in 72% of the cases and in concomitance with *KRAS* mutations in four cases [33]. These findings suggest that the epidemiological landscape of *BRAF* and other genetic alterations in NSCLC will be further cleared as new technologies for genetic testing become available for routine diagnostic purposes.

The *ALK* rearrangements are druggable targets in NSCLC patients with specific inhibitors. Considering the 778 patients examined for both *ALK* rearrangements and *cMET* amplifications, we found that 8% of them harboured *ALK* or *cMET* genetic alterations. The rates of *ALK* rearrangements (5.3%) and *cMET* amplifications (2.1%) found in our cohort were similar to those reported in the scientific literature [34, 35]. *ALK* translocations are common in young patients with non-smoking history and with no apparent ethnic differences [36]; in our study, they were more frequent in young females, without any association with smoking status. *cMET* gene amplification causes 1st generation EGFR-TKI resistance by activating EGFR-independent phosphorylation of ERBB3 and downstream activation of the PI3K/AKT pathway, providing a bypass mechanism. This redundant activation of ERBB3 permits cells to transmit the same downstream signalling in the presence of EGFR-TKIs. This mechanism involves 5–22% of resistant adenocarcinomas and is not related to that dependent on the EGFR^T790M^ mutation on exon 20 (not searched in this study), which represents approximately 60% of resistance cases [37, 38]. Considering that the incidence of *cMET* amplifications in our cohort was 2.1%, most of them seem to occur in subsequent phases of the disease and during treatment with TKIs. This dictates the need for a double inhibition of both EGFR and cMET to overcome the development of drug resistance.

*cMET* was amplified in all four cases in which two concomitant driver genetic alterations were found. Two of them harboured an *EGFR* mutation and a *cMET* amplification, while the remaining two cases presented an *ALK* rearrangement with a simultaneous *cMET* amplification. Indication for a starting therapy combining inhibitors of both altered pathways may be necessary in those cases. No coexistence of *EGFR, KRAS*, or *BRAF* mutations was detected in our cohort, confirming the widely described mutually exclusive mutational pattern. The concomitant *EGFR*-*KRAS* mutations are described mainly in case reports; in a large cohort Chinese study on 5125 patients, 153 cases harbouring concomitant aberrations were found, and among them, 30 carried concomitant *EGFR*-*KRAS* mutations [39]. Nevertheless, recent large cohort studies report a higher grade of the concomitance of *ALK* mutations in NSCLC patients, especially those harbouring *EGFR* mutations [40–43]. *ALK* mutations are reported to occur in concomitance with *EGFR* mutations in 0–6% of cases [40–43]; in our cohort, no such cases were found. Lee et al. analysed the clinical features of six patients harbouring *EGFR*-*KRAS* mutations and six patients with *EGFR*-*ALK* mutations, evidencing different morphological features of the tumours and behaviour to treatments [44]. Most *EGFR*-*KRAS* mutation patients showed papillary and acinar histologic patterns with hobnail cells, while all *EGFR*-*ALK* mutation patients showed solid or cribriform patterns, and three had signet ring cells. Responses to treatment in patients with genetic co-alterations were recently evaluated in a large cohort Chinese study including 3774 cases [45]. The authors reported 63 (1.7%) samples with more than one driver gene mutation; among these, 43 were co-alterations with an *EGFR* mutation, and 20 had an *ALK* rearrangement. In this study, 1st-line EGFR-TKI treatment did not significantly improve the progression-free survival (PFS) of patients harbouring concomitant *EGFR* mutations compared to patients harbouring a single *EGFR* mutation. However, for concomitant *EGFR* mutation patients, TKI therapy was more effective than chemotherapy (median PFS of 10.8 vs 5.2 months, *P* = 0.023) [43]. In any case, the interaction of concomitant genetic alterations in terms of synergism versus the possible dominance of one rather than the other oncogene and the subsequent impact on targeted therapies are currently not completely clarified.

Our study has some limitations, mainly the non-homogeneous distribution of the genetic analyses performed; this simply depended on the gradual introduction of such analyses in clinical practice and the availability of sample tissues for testing. Furthermore, analyses did not include the T790 M mutation on exon 18 or the histological subtypes of the tumours examined. Nevertheless, the consistent number of the global cases analysed taken from real-life clinical practice, the genetic homogeneity of the population examined, and the quality of the methods employed for the tests represent the strengths of our work.

## Conclusions

Our data showed that *KRAS* mutations are the most common genetic alterations in Sardinian patients with lung adenocarcinoma, involving 22.1% of the cases examined and being mutually exclusive with the *EGFR* mutations, which were found in 12.6% of the cases studied. *BRAF* mutations, *ALK* rearrangements, and *cMET* amplifications were detected in 3.2, 5.3, and 2.1% of them, respectively; these figures are relatively low in comparison with most studies in other Caucasian populations. Concomitant mutations were detected only in a few cases, suggesting that they rarely may represent a factor of drug resistance in Sardinians with lung adenocarcinoma, as opposed to other populations in which such concomitance is more common. The low incidence of concomitant *cMET* amplifications at diagnosis suggests that these alterations are acquired in subsequent phases of the disease, often during treatment with TKIs.

## Supplementary information

**Additional file 1: Table S1.** Sequence variations in candidate genes.

## Data Availability

The datasets used and/or analysed during the current study are available from the corresponding author on reasonable request.

## Abbreviations

ALK: Anaplastic lymphoma kinase
BRAF: v-raf murine sarcoma viral oncogene homolog B
EGFR: Epidermal growth factor receptor
FFPE: Formalin-fixed paraffin-embedded
FISH: Fluorescence in situ hybridization
KRAS: Kirsten rat sarcoma viral oncogene homolog
NSCLC: Non-small cell lung cancer
PFS: Progression-free survival
ROS1: ROS proto-oncogene 1
SCLC: Small cell lung cancer
SD: Standard deviation
TKIs: Tyrosine kinase inhibitors
