# Supplementary material for: EGFR, KRAS, BRAF, ALK, and cMET genetic alterations in 1440 Sardinian patients with lung adenocarcinoma
Source: BMC Pulm Med. 2019 Nov 11;19:209. doi: 10.1186/s12890-019-0964-x (PMC6849322; doi:10.1186/s12890-019-0964-x)
Supplement: Supplementary file 1 — Additional file 1: Table S1. Sequence variations in candidate genes. [file 12890_2019_964_MOESM1_ESM.doc]

**Additional file 1: Table S1. Sequence variations in candidate genes**

***EGFR* (183 mutations*)**

| **Mutated cases** | | ***E*xon** | **DNA change** | **Amino Acid Change** | **Effect** | **Designation** |
| --- | --- | --- | --- | --- | --- | --- |
| **No.** | ***%*** |
| 3 | 1.6 | 18 | c.2156 G>C | p.Gly719Ala | Missense | G719A |
| 7 | 3.8 | 18 | c.2155 G>A | p.Gly719Ser | Missense | G719S |
| 1 | 0.5 | 18 | c.2159 C>T | p.Ser720Phe | Missense | S720F |
| 53 | 29.0 | 19 | c.2235-2249del15 | p.746_750del | Deletion, in-frame | *del*ELREA |
| 1 | 0.5 | 19 | c.2235_2249del15 >C | p.E746_A750del>P | Complex - Deletion, in-frame | *del*ELREA>P |
| 1 | 0.5 | 19 | c.2235_2249del15 >ACT | p.E746_A750del>T | Complex - Deletion, in-frame | *del*ELREA>T |
| 6 | 3.3 | 19 | c.2237_2251del15 | p.E746_T751del>A | Complex - Deletion, in-frame | *del*ELREAT>A |
| 5 | 2.7 | 19 | c.2237_2255del18 >TT | p.del746_S752>V | Complex - Deletion, in-frame | *del*ELREATS>V |
| 2 | 1.1 | 19 | c.2235_2249del15 | p.K745_A750del | Deletion, in-frame | *del*KELREA |
| 1 | 0.5 | 19 | c.2239_2251 del13; 2253_2254insA | p.L747_A750del | Deletion, in-frame | *del*LREA |
| 3 | 1.6 | 19 | c.2238_2247G>C | p.L747_A750>P | Complex - Deletion, in-frame | *del*LREA>P |
| 3 | 1.6 | 19 | c.2239_2253del15 | p.L747_T751del | Deletion, in-frame | *del*LREAT |
| 1 | 0.5 | 19 | c.2235_2253del18 | p.L747_T751del>V | Complex - Deletion, in-frame | *del*LREAT>V |
| 4 | 2.2 | 19 | c.2239_2256del18 | p.L747_S752del | Deletion, in-frame | *del*LREATS |
| 9 | 4.9 | 19 | c.2240_2257del18 | p.L747_P753>S | Complex - Deletion, in-frame | *del*LREATSP>S |
| 2 | 1.1 | 19 | c.2252 C>G; 2253_2277del24 | p.T751S; S752_I759del | Complex - Deletion, in-frame | *del*SPKANKEI |
| 1 | 0.5 | 19 | c.2239_2240 TT>CC | p.Leu747Pro | Missense | L747P |
| 1 | 0.5 | 21 | c.2572 C>A | p.Leu858Met | Missense | L858M |
| 70 | 38.3 | 21 | c.2573 T>G | p.Leu858Arg | Missense | L858R |
| 9 | 4.9 | 21 | c.2582 T>A | p.Leu861Gln | Missense | L861Q |

**two patients presented two mutations (delELREA+L861Q and delELREAT>A+L858R)*

*Table continued*

***KRAS* (231 mutations)**

| **Mutated cases** | | ***E*xon** | **DNA change** | **Amino Acid Change** | **Effect** | **Designation** |
| --- | --- | --- | --- | --- | --- | --- |
| **No.** | ***%*** |
| 4 | 1.7 | 2 | c.35G>C | p.Gly12Ala | Missense | G12A |
| 92 | 39.8 | 2 | c.34 G>T | p.Gly12Cys | Missense | G12C |
| 32 | 13.9 | 2 | c.35 G>A | p.Gly12Asp | Missense | G12D |
| 1 | 0.4 | 2 | c.34_35 GG>CT | p.Gly12Leu | Missense | G12L |
| 6 | 2.6 | 2 | c.34 G>C | p.Gly12Arg | Missense | G12R |
| 9 | 3.9 | 2 | c.34 G>A | p.Gly12Ser | Missense | G12S |
| 38 | 16.5 | 2 | c.35 G>T | p.Gly12Val | Missense | G12V |
| 11 | 4.8 | 2 | c.38 G>A | p.Gly13Asp | Missense | G13D |
| 4 | 1.7 | 2 | c.37 G>C | p.Gly13Arg | Missense | G13R |
| 2 | 0.9 | 2 | c.37 G>A | p.Gly13Ser | Missense | G13S |
| 17 | 7.4 | 3 | c.183 A>C | p.Gln61His | Missense | Q61H |
| 5 | 2.2 | 3 | c.183 A>T | p.Gln61His | Missense | Q61H |
| 10 | 4.3 | 3 | c.182 A>T | p.Gln61Leu | Missense | Q61L |

***BRAF* (34 mutations)**

| **Mutated cases** | | ***E*xon** | **DNA change** | **Amino Acid Change** | **Effect** | **Designation** |
| --- | --- | --- | --- | --- | --- | --- |
| **No.** | ***%*** |
| 32 | 94.1 | 15 | c.1799 T>A | p.Val600Glu | Missense | V600E |
| 2 | 5.9 | 15 | c.1799_1800 TG>AA | p.Val600Glu | Missense | V600E |
